# Psychomotor Skills Activities in the Classroom from an Early Childhood Education Teachers’ Perspective

**DOI:** 10.3390/children9081214

**Published:** 2022-08-12

**Authors:** Jorge Rojo-Ramos, María José González-Becerra, Santiago Gómez-Paniagua, Jorge Carlos-Vivas, Ángel Acevedo-Duque, José Carmelo Adsuar

**Affiliations:** 1 Health, Economy, Motricity and Education (HEME) Research Group, University of Extremadura, Avda. de la Universidad s/n, 10003 Cáceres, Spain; 2 BioẼrgon Research Group, University of Extremadura, 10003 Cáceres, Spain; 3 Promoting a Healthy Society Research Group (PHeSo), Faculty of Sport Sciences, University of Extremadura, 10003 Caceres, Spain; 4 Public Policy Observatory, Faculty of Business and Administration, Universidad Autónoma de Chile, Santiago 7500912, Chile

**Keywords:** psychomotor, early childhood education, children

## Abstract

Psychomotricity is a tool that allows the development of different capacities, skills and corporal abilities of people. Currently, it is included in early childhood education programmes due to its importance in children’s development, but, even so, it is not always given the role they deserve. Thus, this study aimed to evaluate the perceptions of early childhood education teachers towards the needs and current state of psychomotor skills in the educational context of Extremadura schools and compare the information provided by teachers that work in rural and urban areas. A questionnaire was administered using a tablet and a Google Forms application. The sample consisted of 216 teachers, selected using a non-probability sampling method based on coexistence sampling. The Mann–Whitney U test was applied to determine the relationships between the different items and dimensions according to the location of the school, and Spearman’s Rho test was used to find out if there is any relationship between the age of the teachers and their responses in the different dimensions. The results showed that psychomotor skills do not receive the place they deserve, with insufficient material and facilities, inadequate training, few sessions and inappropriate programming, together with the rest of the components of the cycle. Therefore, it can be concluded that it is necessary to include psychomotor skills in the training of teachers and that the centres should be concerned about providing teachers with the necessary material and spaces for their work.

## 1. Introduction

Nowadays, it is increasingly common to find children with motor skills deficits, not only at an early age, but also throughout their primary school years, especially those who have never participated in out-of-school physical activity [1]. The result of good or bad motor skills may be due to reasons such as lack of interest of the children or even that the school does not give it enough importance [2].

These problems not only affect motor skills but also have an impact on cognition, impairing the ability of children to process information from everything they perceive. Going hand in hand with the terms cognition and motor skills, psychomotricity arises, which is defined as a discipline that considers the person as a whole and synthesizes, therefore, motor skills and the psyche [3]. Conversely, psychomotor skills are a technique that influences the intentional act to stimulate or modify it by using bodily activity, or are an approach to educational intervention that aims to develop motor, expressive and creative possibilities through the body [4,5]. Psychomotor skills provide benefits, such as facilitating the acquisition of the body schema, addressing different motor patterns, promoting body control, affirming laterality, developing balance and creating learning habits or social integration [3,6]. Hence, psychomotor education guarantees the development of intelligence through motor action, constituting a preventive educational action. Consequently, it is necessary that the teacher has training or knowledge in psychomotor skills and puts them into practice from early childhood education because psychomotor aspects will contribute positively to the student’s learning [7]. Alves, Lussac, Fonseca and others [8,9,10] agree that psychomotor skills promote learning and the overall development of the child in a simplified and evolutionary way. Thus, they emphasize the importance of the teacher having training or specialization in psychomotor skills and acting as a facilitator, transmitting his knowledge to the students and putting psychomotor activities into practice from preschool, whilst also considering that the family is of enormous importance.

During early childhood education, educators observe different perceptual and motor possibilities: the identification of sensations, the global and partial knowledge of the body or different expressive possibilities of the body through psychomotricity [11]. Yáñez et al. [12] state that the confusion between this term and Physical Education is caused by the lack of knowledge of the current model of Physical Education and the ambiguity of the meaning of the term “psychomotricity”. Another study, focused on the perception of teachers in this educational stage [13], reflects the reality of content with little educational weight, either because of the little importance given to early childhood education or psychomotor skills in society. Many of these teachers understand the importance of this content, despite the fact that in most schools these sessions are relegated to the background, with only one hour per week dedicated to them [14]. To this is added the belief that they do not have objective tools to acquire reliable information on the psychomotor development of their students [15], which is a fundamental aspect of the planning and achievement of objectives in this area [16], since the primary condition for promoting these psychomotor programmes is to allow teachers to determine their own needs and possibilities [17].

Similarly, teachers’ training is a major factor in any of the areas that encompass education at any level [18]. Teacher education helps the enrichment of educational processes by immersing students in activities that provide meaningful and constructivist learning, resulting in a holistic and global stimulation for the children [19]. However, previous literature has already pointed out the deficit of content in the area of psychomotor skills during university teaching in early childhood education, as compared to those developed during a primary education degree [4,20]. In addition, postgraduate teacher training is perceived by students as deficient and of low quality [21], so these contents are normally implemented by specialists in physical education at primary school level [22].

Due to all these benefits of psychomotor skills, in populations of different ages and conditions, this study aims to evaluate the perceptions of early childhood education teachers towards the needs and current state of psychomotor skills in the educational context in Extremadura schools. We also aim to compare the information provided by teachers that work in a rural location centre compared to those who work in an urban environment. We choose the first educational stage because psychomotor skills appear in the educational curriculum as important, and we believe it is a crucial stage in the child’s development. Based on previous research [23], we hypothesize that early childhood education teachers will not be sufficiently prepared and will not receive adequate resources to teach the contents established in the educational curriculum on psychomotricity.

## 2. Materials and Methods

### 2.1. Participants

Two-hundred and sixteen second-cycle pre-school education (3 to 6 years old) teachers (82.9% females and 17.1% males) from public schools in Extremadura were selected using a non-probabilistic sampling method based on coexistence sampling [24]. Regarding the location of the educational centres, 35.6% and 64.4% were rural and urban schools, respectively. The mean age of the sample was 43.94 (9.80) years, and their mean experience was 18.08 (9.50) years.

Table 1 shows the distribution of the participants according to sex, centre location, course taught, age and teaching experience.

### 2.2. Instruments and Measures

Sociodemographic data were obtained through a questionnaire that included five questions: gender, grade in which they teach, school environment, age and teaching experience.

The Questionnaire on Psychomotricity in the Educational Context (CPCE) [23] was used. This instrument is composed of a total of 19 items grouped into six dimensions: (1) Training (items 1 and 2), which measures the training received by the teacher, both previous and current, in child psychomotor skills; (2) Programming (items 3 to 6), which refers to the organization and approach to psychomotor activities, considering key aspects such as the characteristics and daily development of the students, the different motor contents or the perceptions of other teachers; (3) Material (items 7 to 10), which refers to the availability and adequacy of the material available/used in psychomotor classes to the students’ characteristics; (4) Personnel (items 11 and 12), regarding the competence of the teachers present in the centre to develop the psychomotor skills content in an adequate manner; (5) Contents (items 13 to 15), defining the fact that the psychomotor teaching programme covers all relevant aspects of psychomotor skills; and (6) Sessions (items 16 to 19), in reference to the characteristics of the classes taught in psychomotor skills in the educational context that allow the psychomotor development of students. The instrument uses a 5-level Likert scale from 1, “totally disagree” to 5, “totally agree”. This questionnaire was previously validated on early childhood education teachers in another region of Spain (Murcia) [23]. Nevertheless, the reliability outcomes for each CPCE questionnaire dimension, based on our data, were the following: “formation” = 0.75, “programming” = 0.83, “material” = 0.77, “staff” = 0.85, “contents” = 0.79 and “sessions” = 0.83; which means satisfactory values according to Nunnally and Bernstein (1994) recommendations because all values are above 0.70 [25].

### 2.3. Procedures

It was decided to use the Google Forms application to prepare the sociodemographic and CPCE data questionnaires. It allowed us to save costs and facilitate both the delivery of the questionnaires to the participants and to store the responses in the same database [26]. Data collection was carried out between September and December 2021.

To access the sample, we used the database of public schools in the Autonomous Community of Extremadura belonging to the Department of Education and Employment of the Regional Government of Extremadura and selected the contact details of the schools in which preschool education is taught.

After that, all the selected centres were contacted through an e-mail addressed to the Early Childhood Education teachers informing them about the study and indicating the URL for accessing the form and the informed consent.

Due to the response rate not being sufficient during the first month, it was decided to resend the e-mail and make a telephone call to the centre informing them of the study and the procedures for collaborating on it. Thus, the sample was increased until the necessary data were obtained.

### 2.4. Statistical Analysis

Data collection analysis was performed with the Statistical Package for Social Sciences (SPSS) version 23.0 for MAC. The Kolmogorov–Smirnov test was used to analyse the normality and homogeneity of data. Results indicated that data was not normal-distributed, so non-parametric tests were applied. Thus, the Mann–Whitney U test was executed to analyse the relationships between the different CPCE items and dimensions according to the location of the centre, and Spearman’s Rho correlation test was used to check the relationship between each dimension and teaching experience. Correlation thresholds were interpreted as follow [27]: 0.01–0.09, negligible; 0.20–0.29, weak; 0.30–0.39, moderate; 0.40–0.69, strong; and ≥0.70, very strong. Finally, Cronbach’s alpha was used to calculate the reliability of each instrument dimension. Alpha level was set at *p* ≤ 0.05.

## 3. Results

Table 2 shows the associations between the different items of CPCE based on the centre location. Overall, significant differences (*p* < 0.01) were observed between rural and urban centres, except in items 10, 11, 14 and 15, which referred to the importance of materials and the safety and space adaptation to children’s characteristics, the presence of a psychomotor specialist at school and the general objective of psychomotor skills.

Table 3 presents the scores of each CPCE dimension according to the centre location. Results showed significant differences between rural and urban centres in all dimensions (*p* < 0.01), except for “contents” (*p* = 0.48). Specifically, urban centre teachers reported higher scores in “training”, “programming”, “material” and “sessions” dimensions than their counterparts from rural centres. Slightly higher outcomes in the “personal” dimension were also observed in urban centre teachers compared to rural centre teachers.

Table 4 shows the correlations between factors and age. Overall, no significant associations were observed between age and dimensions, except for “programming” (*rho* = 0.27; *p* < 0.01) and “material” (*rho* = 0.28; *p* < 0.01) dimensions, which were weakly and positively associated with age.

## 4. Discussion

This research arose from the need to find out whether psychomotor skills are really being given importance in early childhood education. For this purpose, the CPCE questionnaire was used to clarify whether teachers have received adequate training, carry out a correct programme, have the necessary personnel and material and use appropriate content in the planned number of sessions.

The main findings regarding the training received by teachers revealed that they never, or almost never, received the correct training in psychomotor skills. These findings coincide with those shown by Díaz and Sosa [21], who reported that teachers do not have the necessary training in the psychomotor field due to insufficient subjects throughout their careers. Similarly, Solís-Picatto et al. [28] concluded that teachers do not have specific training in psychomotor skills; however, these authors indicated that, despite this, teachers are trained to carry out the sessions. Nevertheless, it can lead to a routine of sessions without specific objectives that may harm the evolution of the child. Other authors [29,30] detected that teachers demanded training in psychomotor skills, as well as the need for training and updating courses in psychomotor skills. If we analyse these findings based on centre location, we observe that rural environments present lower scores than urban environments.

Concerning the programming dimension, our findings highlight that rural environment centres always reported close to never, or almost never, outcomes, in contrast to the urban centres which show always, or almost always, scores. The results obtained are like those presented in previous studies [31,32], in which teachers programmed their activities daily according to individual characteristics, but they did not follow a programme shared with other teachers. This highlights the need to integrate psychomotor education into the curriculum and be programmed systematically like the rest of the areas. Moreover, it would be important to consider that previous literature on rural education revealed inadequacies in programming due to a lack of funding, including fewer specialists, unskilled employees, restricted resources and fewer programme options [33,34].

Regarding personnel and material dimensions, we can affirm that the scores are generally very low in both urban and rural centres. Thus, teachers agree on the need for the safety of the space and the lack of specialists being the only dimensions among which we do not find significant differences, since the availability of materials and space and the need for adequate training differ in the answers according to the type of centre due to the presence of more resources available in urban centres. Our findings coincide with those shown by other authors [35,36,37], among whom some express limitations among teachers when considering a degree necessary and the importance and need for adequate conditioning of materials and spaces. They consider it the main reason for not carrying out psychomotor skills sessions since they play a central role in students’ motivation. The lack of adapted infrastructures for psychomotor work was also reported previously by Pons Rodríguez and Arufe-Giráldez [14].

In terms of content, most of the teachers surveyed agree that psychomotricity is physical education in early childhood education, and that its general objective is to develop motor and psychological skills independently of the environment, except for defining psychomotricity as physical education. This conceptual–epistemological confusion between the terms physical education and psychomotricity has been previously documented in the literature [12].

Finally, regarding sessions dimension, the adequacy of the number of sessions, their duration and the work methodology in psychomotor classes is similar in general, although there are differences between rural centres where the number of sessions and their duration are almost never adequate compared to urban centres where they tend to be more adjusted. It could be explained by the previous finding reported by Alonso-Álvarez and Pazos-Couto [31] who highlighted that the importance given to psychomotor skills is low because the work in the classroom is little. Thus, it is necessary to think about how the time dedicated to psychomotor skills practice and its distribution is spent, even more so considering that previous research has shown that distributed practice is more effective in the development of psychomotor skills than massed practice [38,39,40], i.e., short frequent practice sessions are more effective than practice over a long period of time.

To sum up, urban and rural centres differ in all dimensions except for contents. The differences are always in the same way; i.e., rural centres find more difficulties when it comes to having valid materials or facilities, trained personnel, adequate content or sessions than urban centres, as it was previously reported [41,42]. Observing correlations between the different dimensions according to age, the correlations are significant in the programming and material dimensions, so it can be said that the score received by these dimensions will vary according to the age of the respondents. On the contrary, the rest of the dimensions will not vary according to age. It may be due to experienced educators who are more trained and more comfortable and motivated with psychomotricity contents. Moreover, older educators invert on more materials. However, future research is needed for clarifying this point.

Considering all the above, there are evident needs, from the teachers’ point of view, in terms of previous and ongoing training as well as in the competence of the teachers themselves, to develop adequate psychomotor skills sessions. In the same way, teachers who carry out their teaching labour in rural centres report scores lower than those in urban environments, except in the last dimension of the questionnaire. Therefore, public and private organizations must determine and develop appropriate educational content to promote the training of teachers at any professional stage, providing them with useful tools so that society can enjoy the innumerable benefits of this content. In the same sense, new strategies should be developed to improve the situation of rural centres since it is evident that they lack resources in terms of psychomotor content, which could be dangerous due to the lower number of stimuli and psychomotor opportunities in these populated areas. As the main limitations of the present study, we note the lack of previous research on the topic in question, the subjective data and the lack of generalizability because this study focuses on a specific region of Spain. Future studies should be oriented to assess the impact of more training of teachers on the children’s development, considering that more psychomotor experience in preschool classes may enhance language development and other aspects of cognition, as is reported in previous literature. Moreover, it should be valued the feasibility to modify the curriculum development, highlighting the importance of play in the learning and practising of new skills beyond the health reasons to make exercise available to children, considering the importance of play and skills in the preschool stage. Moreover, the inclusion of teachers from other regions and countries should be considered to compare the different points of view, depending on the importance that the educational curriculum gives to psychomotricity.

## 5. Conclusions

The early childhood education teachers surveyed in the community of Extremadura agree that they do not have the necessary material and facilities for teaching psychomotor skills and do not have adequate staff or training. Moreover, they agree that they programme the classes and adapt them to the motor content and individual characteristics, but this occurs mainly in urban centres, being almost non-existent in rural environments. The only drawback is that the programming is carried out individually and not in an organized manner among the teachers who share a cycle. The number of sessions is considered adequate by teachers in urban centres but deficient in rural centres.

The only dimension that receives high scores and with which teachers agree is the objectives of psychomotor skills, although many confuse it with a type of physical education in early childhood education. The age of the teachers was not a determining factor in most of the responses but it does influence the programming and the material and facilities.

## Figures and Tables

**Table 1:** Distribution of the sample (*N* = 216).

Variable	Categories	*N*/M	%/SD
Gender	Male	37	17.1
	Female	179	82.9
Course	First	60	27.8
	Second	43	19.9
	Third	113	52.3
Centre Environment	Rural	77	35.6
	Urban	139	64.4
Age (years)		43.94	9.80
Teaching experience (years)		18.08	9.50

M: mean; SD: standard deviation.

**Table 2:** Descriptive statistics and differences in CPCE questionnaire items based on centre location.

	Centre location			
Item	Total	Rural	Urban	
	M (SD)	M (SD)	M (SD)	*p*
1. The specific training received on child psychomotor skills is adequate.	2.28 (0.73)	1.62 (0.65)	2.64 (0.48)	<0.01 **
2. I have taken training and refresher courses on psychomotor skills.	2.05 (1.03)	1.55 (0.83)	2.33 (1.02)	<0.01 **
3. I program the daily psychomotor activities that I carry out with my students.	3.28 (1.24)	2.22 (1.38)	3.86 (0.61)	<0.01 **
4. I adapt the psychomotor programming to individual characteristics.	3.17 (1.42)	2.19 (1.62)	3.71 (0.95)	<0.01 **
5. I divide the objectives of the psychomotor subject according to the different motor aspects.	3.44 (1.42)	2.30 (1.71)	4.08 (0.63)	<0.01 **
6. I carry out the psychomotor programming together with the other colleagues of the cycle.	1.72 (0.93)	1.36 (0.56)	1.92 (1.03)	<0.01 **
7. I have the necessary material to be able to carry out my psychomotor programming.	2.16 (0.90)	1.47 (0.68)	2.55 (0.77)	<0.01 **
8. I consider the material used in the development of a psychomotricity session to be decisive.	3.12 (0.67)	2.90 (0.50)	3.24 (0.72)	<0.01 **
9. I have the necessary space to carry out the psychomotricity sessions in an adequate way.	2.21 (0.92)	1.43 (0.63)	2.64 (0.77)	<0.01 **
10. It is important that the materials and the space used in the psychomotor sessions are safe and adapted to the needs of the children.	4.98 (0.19)	4.97 (0.22)	4.99 (0.17)	0.67
11. The school has a psychomotor specialist to work with children.	1.87 (1)	1.70 (0.60)	1.96 (1.15)	0.56
12. My colleagues, who teach psychomotor skills, have adequate training.	2.27 (0.94)	1.82 (0.68)	2.52 (0.98)	<0.01 **
13. Psychomotricity is physical education in Early Childhood Education.	4.67 (0.47)	4.79 (0.40)	4.60 (0.49)	<0.01 **
14. The general objective of psychomotor skills is the development of motor skills.	4.77 (0.42)	4.71 (0.45)	4.80 (0.40)	0.16
15. The general objective of psychomotricity is the development of psychological skills.	4.59 (0.49)	4.62 (0.48)	4.58 (0.49)	0.49
16. The number of weekly psychomotor skills sessions is sufficient for developing the contents.	2.87 (1.06)	2.17 (1.17)	3.26 (0.75)	<0.01 **
17. The duration of the psychomotor session is sufficient for the child’s psychomotor development.	3.05 (1.21)	2.22 (1.07)	3.51 (1.03)	<0.01 **
18. I conduct at least two weekly sessions of psychomotricity in same classroom.	3.89 (1.36)	2.68 (1.57)	4.57 (0.49)	<0.01 **
19. I believe that working with children on psychomotor skills in a directed way, rather than in an experiential way, is more effective in achieving the proposed objectives.	3.03 (1.23)	2.43 (1.32)	3.37 (1.05)	<0.01 **

Note: M = mean value; SD = standard deviation. Note: The correlation is significant in ** *p* < 0.01. Each dimension score is based on a Likert scale (1–5).

**Table 3:** Descriptive analysis of each dimension of the CPCE questionnaire.

		Location		
Dimensions	M (SD)	Rural	Urban	*p*
Formation	2.16 (0.80)	1.58 (0.69)	2.48 (0.66)	<0.01 **
Programming	2.90 (1.03)	2.01 (1.19)	3.39 (0.46)	<0.01 **
Material	3.11 (0.56)	2.69 (0.37)	3.35 (0.51)	<0.01 **
Staff	2.06 (0.91)	1.75 (0.61)	2.24 (1)	<0.01 **
Contents	4.67 (0.39)	4.71 (0.36)	4.65 (0.40)	0.48
Sessions	3.21 (1)	2.37 (1.10)	3.67 (0.53)	<0.01 **

Note: M = mean value; SD = standard deviation. The correlation is significant in ** *p* < 0.01. Each dimension score is based on a Likert scale (1–5).

**Table 4:** Correlations between CPCE dimensions and the variable years of experience.

Dimensions	Age ρ (*p*)
Formation	−0.02 (0.68)
Programming	0.27 (<0.01 **)
Material	0.28 (<0.01 **)
Staff	−0.07 (0.26)
Contents	0.05 (0.43)
Sessions	−0.01 (0.93)

Note: Correlation is significant at ** *p* < 0.01. Each score obtained in the dimensions is based on a Likert scale (1–5).

## Data Availability

The datasets used during the current study are available from the corresponding authors on reasonable request.
